# RNA sequencing read depth requirement for optimal transcriptome coverage in *Hevea brasiliensis*

**DOI:** 10.1186/1756-0500-7-69

**Published:** 2014-02-01

**Authors:** Keng-See Chow, Ahmad-Kamal Ghazali, Chee-Choong Hoh, Zainorlina Mohd-Zainuddin

**Affiliations:** 1 Biotechnology Unit, Malaysian Rubber Board, Rubber Research Institute of Malaysia, Experiment Station, Kuala Lumpur 47000, Sungai Buloh, Selangor, Malaysia; 2 Codon Genomics SB, No. 26, Jalan Dutamas 7, Taman Dutamas, Balakong 43200, Seri Kembangan Balakong, Selangor, Malaysia

**Keywords:** Transcriptome, RNA-Seq, Sequencing, *Hevea brasiliensis*, Rubber tree, *de novo* assembly, Gene transcript

## Abstract

**Background:**

One of the concerns of assembling *de novo* transcriptomes is determining the amount of read sequences required to ensure a comprehensive coverage of genes expressed in a particular sample. In this report, we describe the use of Illumina paired-end RNA-Seq (PE RNA-Seq) reads from *Hevea brasiliensis* (rubber tree) bark to devise a transcript mapping approach for the estimation of the read amount needed for deep transcriptome coverage.

**Findings:**

We optimized the assembly of a *Hevea* bark transcriptome based on 16 Gb Illumina PE RNA-Seq reads using the Oases assembler across a range of k-mer sizes. We then assessed assembly quality based on transcript N50 length and transcript mapping statistics in relation to (a) known *Hevea* cDNAs with complete open reading frames, (b) a set of core eukaryotic genes and (c) *Hevea* genome scaffolds. This was followed by a systematic transcript mapping process where sub-assemblies from a series of incremental amounts of bark transcripts were aligned to transcripts from the entire bark transcriptome assembly. The exercise served to relate read amounts to the degree of transcript mapping level, the latter being an indicator of the coverage of gene transcripts expressed in the sample. As read amounts or datasize increased toward 16 Gb, the number of transcripts mapped to the entire bark assembly approached saturation. A colour matrix was subsequently generated to illustrate sequencing depth requirement in relation to the degree of coverage of total sample transcripts.

**Conclusions:**

We devised a procedure, the “transcript mapping saturation test”, to estimate the amount of RNA-Seq reads needed for deep coverage of transcriptomes. For *Hevea de novo* assembly, we propose generating between 5–8 Gb reads, whereby around 90% transcript coverage could be achieved with optimized k-mers and transcript N50 length. The principle behind this methodology may also be applied to other non-model plants, or with reads from other second generation sequencing platforms.

## Background

Transcriptome analysis has become increasingly powerful through advances in second generation sequencing technologies from companies such as Illumina, Roche and Life Technologies. Improvements in sequencing chemistry and read length have enabled unprecedented depth of sequencing, limited only by cost and availability of biological material. In particular, RNA sequencing (RNA-Seq), also known as whole transcriptome shotgun sequencing, has emerged as a valuable tool for profiling expressed genes in plants and other organisms [1-3]. The depth of transcriptome sequencing provided by RNA-Seq has thus provided a cost-effective means of qualitative and quantitative analyses of gene transcripts in many non-model plant species including the rubber tree, *Hevea brasiliensis*, the subject of the present study.

Parallel with progress in sequencing technologies, numerous softwares have been developed to assemble *de novo* transcriptomes. Among the most commonly used sofwares are Velvet [4], Oases [5], SOAPdenovo [6], ABySS [7], Trinity [8], MIRA [9], Newbler (Roche) and CLC (CLC bio). In the absence of a reference genome, the assemble-then-align approach is used in place of the align-then-assemble approach [10-12]. Additional procedures are often integrated in order to improve the quality of the *de novo* transcriptome assembly. This includes weighing the relative merits of more than one assembler [13-21], optimizing transcript numbers and lengths across different k-mers and other assembly parameters [16,17,22-26], hybrid assembly of data from different sequencing platforms [21,27-30] and alignment of transcripts to sequences from the same or related species [18,30-32].

Challenges in *de novo* transcriptome assembly in higher plants lie in the immense number of gene transcripts, large variations in transcript expression levels, presence of alternatively spliced transcript variants and issues in strand directionality [11,12]. Owing to such problems, *de novo* assembly requires significantly greater sequencing depth as compared with reference-based assembly. Especially in the case of non-model plants, few guidelines are available for determining the amount of reads to generate to enable deep coverage of transcripts expressed in a particular sample. The general practice commonly adopted, especially for new entrants in second generation sequencing, is to piggy-back on ballpark estimates adopted for the model species. From a survey of recent publications on *de novo* transcriptome analysis in non-model plants, the read generation per sample could fall below 100 Mb or it could be high as 7 Gb, with 2–5 Gb being the most common sequencing depths [13,18,19,21,23,26-49]. Therefore, there is need for a practical procedure to estimate the reads needed for deep coverage of gene transcripts in *de novo* assembly where such information is unavailable.

*Hevea brasiliensis* is the commercial source of natural rubber (cis-polyisoprene). The tree, a diploid species (2n = 36) from the Euphorbiaceae family, has a C value of about 2 pg as estimated by flow cytometery [50]. Compared to model plants, transcript resources in this economically important species have only been initiated relatively recently, with the analysis of Expressed Sequence Tags (ESTs) from latex [29,51-54]. The first crop of publications on the application of second generation sequencing in *Hevea* rubber genomics emerged in 2011 (Table 1). In these papers, functional profiling of gene transcripts was reported in latex, leaf and bark tissues, focussing on rubber biosynthesis, molecular markers and transcription factors [29,55-60]. Overall, the depth of sequencing reported in these papers was between 0.2-5 Gb per sample based on Illumina PE-RNA-Seq or 454 pyrosequencing platforms (Table 1). *Hevea* being a tree, analysis of its gene expression is often in RNAs prepared from distinct cells, tissues or organs, including RNAs from the same sample types but under different physiological conditions [61-64]. Having a means of assessing the degree of coverage of genes that are expressed in various samples would be important to achieve a normalized set of *Hevea* gene transcripts. In this paper, we describe a method to estimate datasize requirement for high transcriptome coverage based on an analysis of assembly statistics of Illumina PE RNA-Seq reads generated from *Hevea* bark. We first optimized and validated a 16 Gb bark assembly across a range of datasizes and k-mers. Subsequently, we applied a transcript mappability method to illustrate the trend of gene transcript coverage by different read amounts that had been assembled using a range of k-mers.

**Table 1:** Publications containing applications of second generation sequencing in rubber tree transcriptome analysis

**No.**	**Publication**	**Year**	**Sequencing method**	**Transcriptome type (length and number of reads)**
1	Xia et al. [55]	2011	PE-RNA-Seq (Illumina)	Latex and leaf combined; clone RY7-33-97 (12 mil. reads or 1 Gb approx.)
2	Pootakham et al. [56]	2011	454 pyrosequencing (Roche)	Information not available
3	Triwitayakorn et al. [57]	2011	454 pyrosequencing (Roche)	Shoot apical meristem; clone RRIM 600 (2 mil. reads or 676.5 Mb approx.)
4	Chow et al. [29]	2012	RNA-Seq (Illumina)	Latex; clone RRIM 600 (10 mil. reads or 350 Mb approx.)
5	Li et al. [58]	2012	PE-RNA-Seq (Illumina)	Bark; clone RY7-33-97 (30 mil. reads or 3 Gb approx.)
6	Duan et al. [59]	2013	454 pyrosequencing (Roche)	Leaf, bark, latex, root, embryogenic tissues; clone PB 260(0.5 mil. reads or 200 Mb approx. per tissue)
7	Rahman et al. [60]	2013	PE-RNAseq (Illumina);	Leaf; clone RRIM 600 (4.89 Gb);
			454 pyrosequencing (Roche)	Leaf; clone RRIM 600 (1,085 Mb)

Sequencing depth, tissue types and the tree clones in each project are indicated.

## Findings

### Generation of *Illumina* PE RNA*-*Seq *Hevea* tissue libraries and *de novo* assembly

The development of an approach to determine the datasize required for deep coverage of a *de novo* transcriptome arose from the generation of three *Hevea* tissue read libraries as part of a programme in developing genomic resources for the rubber tree. Considerably more PE RNA-Seq raw reads were available for bark (16.9 Gb) as compared with latex and leaf tissues (4.9-5 Gb each) (Table 2; see Materials and methods). Hitherto, similar work on the rubber tree involving second generation sequencing (Table 1) has far lower transcriptome coverage per sample compared to what we have generated. Taking cognizance of the immensity of the bark read library, we asked the following questions: (a) What is the trend of assembly characteristics of a significantly larger read library? and (b) Can we devise a method to relate datasize requirement to the degree of coverage of transcripts expressed in the sample?

**Table 2:** **Quality processing of reads from three *Hevea *** **tissue libraries**

**Latex library**	**Read number (forward + reverse)**	**Read size (forward + reverse)**
Raw reads	50,384,572 (100%)	5,038,457,200 (100%)
Clean reads	49,393,389 (98.03%)	4,709,104,798 (93.46%)
Paired reads	48,650,932 (96.56%)	4,647,858,661 (92.25%)
Orphan reads (single end)	742,457 (1.47%)	61,246,137 (1.21%)
**Leaf library**	**Read number (forward + reverse)**	**Read size (forward + reverse)**
Raw reads	49,578,322 (100%)	4,957,832,200 (100%)
Clean reads	47,662,360 (96.14%)	4,512,413,782 (91.02%)
Paired reads	46,062,766 (92.90%)	4,373,106,379 (88.21%)
Orphan reads (single end)	1,599,594 (3.23%)	139,307,403 (2.81%)
**Bark library**	**Read number (forward + reverse)**	**Read size (forward + reverse)**
Raw reads	169,887,626 (100%)	16,988,762,600 (100%)
Clean reads	166,258,828 (97.86%)	15,983,753,737 (94.08%)
Paired reads	163,316,702 (96.13%)	15,726,859,825 (92.57%)
Orphan reads (single end)	2,942,126 (1.73%)	256,893,912 (1.51%)

**Figure 1:**
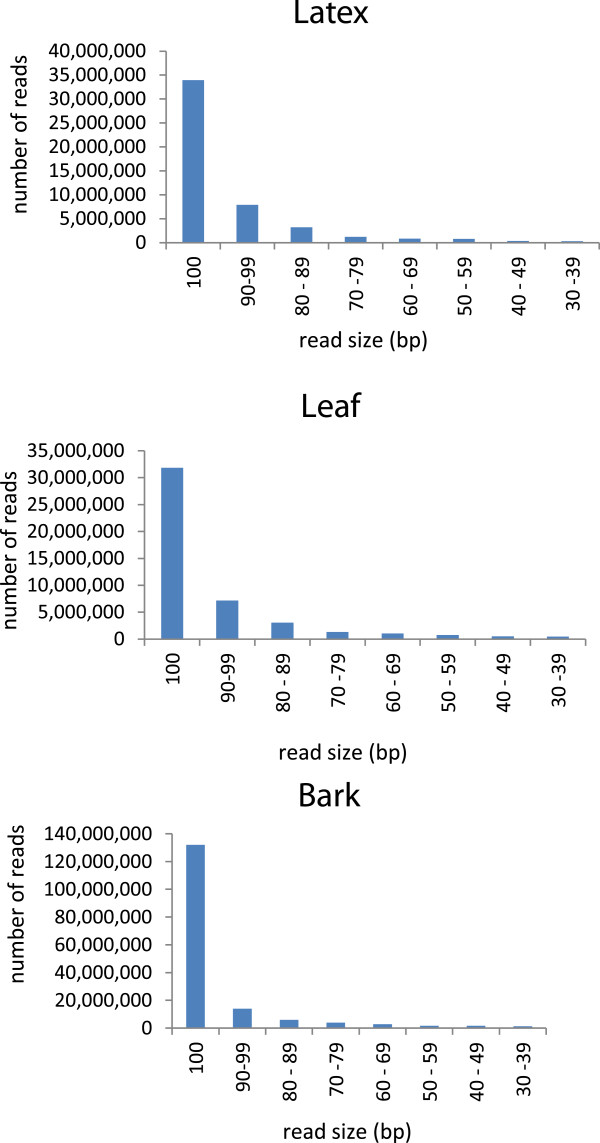
Size distributions of clean paired reads from latex, leaf and bark libraries.

We first processed the bark, latex and leaf raw reads for quality, and found a very high percentage of clean reads (approximately 96-98%) (Table 2; see Materials and methods). The distribution of read length categories also indicated high PE RNA-Seq quality based on the fact that a vast majority of clean paired reads was in the largest size category (100 nt) (Figure 1). Henceforth in this report, the clean paired reads from bark are referred to as the 16 Gb read set while those from latex or leaf as the 5 Gb read set (Table 2). A number of plant *de novo* transcriptome projects have reported the use of multiple k-mers in assembly optimization [13,21,27,65]. Therefore, we analyzed the effect of two parameters on transcriptome assembly, namely read amount and k-mer size.

To do this, the 16 Gb bark read set was assembled in incremental quantums of 1, 3, 5, 8, 10, 13 and 16 Gb using the Oases assembler and a k-mer range of 51–77 (see Materials and methods). As shown in Table 3, the higher the k-mer, the lower was the number of transcripts generated by a particular datasize. At the same time, the larger the dataset size, the greater the number of transcripts generated using a particular k-mer. On the other hand, the transcript N50 length (see Materials and methods) showed a bell shape distribution from k-mer 51 to 77 in all datasize sub-assemblies with the exception of the 1 Gb datasize (N50 peak values are highlighted in Table 3). The k-mer at which the N50 value peaked in this bell shape distribution was thus referred to as the “optimized k-mer” of assembly for a particular datasize (and accordingly the “optimized N50”). As seen in Table 3, the optimized N50 became larger with increasing read depth, and this corresponded also with increment in the size of optimized k-mers. Hence, larger data sizes facilitated not only an increase in the number of transcripts assembled, but also an increase in transcript length. We therefore propose that by a judicious combination of datasize and k-mer range, the ensuing N50 trend may serve as a criterion for determining optimal *de novo* assembly.

**Table 3:** **Statistics of incremental bark assemblies across k**-**mers**

**k-mer size**	**1 Gb**		**3 Gb**		**5 Gb**		**8 Gb**		**10 Gb**		**13 Gb**		**16 Gb**	
	**N50 (bp)**	**Total transcripts**	**N50 (bp)**	**Total transcripts**	**N50 (bp)**	**Total transcripts**	**N50 (bp)**	**Total transcripts**	**N50 (bp)**	**Total transcripts**	**N50 (bp)**	**Total transcripts**	**N50 (bp)**	**Total transcripts**
51	1,389	68,942	1,734	102,352	1,741	131,979	1,695	170,838	1,648	193,885	1,599	224,292	1,542	254,026
53	1,375	64,265	1,763	94,288	1,797	120,998	1,778	157,127	1,741	178,204	1,701	206,355	1,654	234,717
55	1,353	59,325	1,783	86,117	1,832	110,086	1,834	142,292	1,813	161,486	1,798	186,542	1,758	212,283
57	1,343	54,670	1,798	78,651	1,852	100,131	1,872	129,469	1,864	146,550	1,861	169,917	1,828	192,775
59	1,315	50,440	1,799	72,051	1,876	91,351	1,905	117,384	1,901	133,175	1,903	154,642	1,880	176,018
61	1,288	46,261	***1,804***	65,903	1,891	83,027	1,926	106,569	1,933	120,901	1,940	139,498	1,928	159,832
63	1,255	42,626	1,791	60,666	1,889	75,893	1,959	96,676	1,959	109,522	1,969	126,478	1,956	143,857
65	1,225	39,223	1,782	55,645	***1,900***	69,159	1,966	87,780	1,980	98,782	1,989	115,028	1,988	130,546
67	1,199	35,773	1,753	51,333	1,892	63,469	1,970	80,153	1,997	90,055	2,010	104,399	2,025	118,385
69	1,147	32,606	1,731	47,383	1,895	58,112	***1,976***	72,612	2,007	81,560	2,024	94,500	2,043	106,974
71	1,111	29,621	1,691	43,910	1,878	53,357	1,974	66,249	***2,017***	74,220	2,036	85,874	2,061	96,653
73	1,068	26,674	1,652	40,633	1,846	49,412	1,967	60,561	2,010	67,593	2,040	77,667	***2,068***	87,612
75	1,034	23,699	1,606	37,433	1,808	45,312	1,946	55,384	1,997	61,680	***2,041***	70,331	2,066	79,038
77	982	20,746	1,550	34,531	1,770	41,621	1,923	50,636	1,967	56,233	2,022	63,767	2,057	71,504

The peak N50 length from each datasize assembly (other than for 1 Gb) is highlighted in bold italics.

### Validation of the bark transcriptome

In this study, a k-mer of 73 assembled 87,612 transcripts from the 16 Gb read set with a transcript N50 value of 2,068 bp (Table 3). At this stage, this assembly was referred to as the optimized bark assembly for 16 Gb reads. Due to the importance of quality *de novo* assembly, we performed three mapping analyses to validate the 16 Gb bark transcriptome (see Materials and methods). First of all, 255 publicly available *Hevea* cDNAs which had been verified to contain complete open reading frames or ORFs were mapped to 87,612 bark transcripts using the Megablast software. In the absence of more cDNAs containing complete *Hevea* proteins, rubber-specific ORF quality of bark transcripts could only be evaluated using these sequences which also included isoforms for several gene families (see Additional file 1: Table S1). The results showed that 250 *Hevea* ORFs (of 255) had hits to bark transcripts with sequence identity match ranging from 86-100% (Table 4 and Additional file 1: Table S1). Also, 80% of the 250 ORFs showed a minimum of 70% utilization of ORF sequence length in their match alignments with bark transcripts (Table 4 and Additional file 1: Table S1). These observations indicated that a high proportion of transcripts within the optimized bark assembly encoded complete or near-complete *Hevea* proteins.

**Table 4:** **Mapping of 255 *Hevea *** **ORF sequences to transcripts from the optimized 16 Gb bark assembly**

	**Number**	**Percentage**
Total queries (*Hevea* complete ORFs)	255	
Queries with hits to bark transcripts	250	100%
Queries with bark transcript hits where ORF coverage ≥ 70%	200	80%

Secondly, completeness of gene representation in the 16 Gb transcriptome was assessed by mapping 87,612 bark transcripts to a set of 248 core eukaryotic genes (CEGs) that had been shown to be a reliable indicator of completeness of gene space in eukaryotic species [66]. Although initially used to assess gene space in newly sequenced genomes, the approach was recently applied to transcriptomes and it also complements other transcript metrics such as N50 length. Using BlastX, 87,612 bark transcripts detected 247 out of 248 CEG proteins (98%) (e-value ≤ 1e^-10^). Thus, these results support the completeness or depth of bark gene representation in this transcriptome.

**Figure 2:**
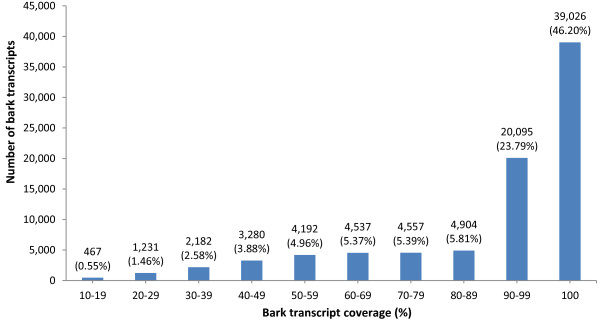
**Number of transcripts from the optimized 16 Gb bark assembly with hits to rubber genome scaffolds.** Hits are classified into transcript-to-scaffold coverage categories describing the extent of alignment from 10-100%. A total of 84,471 bark transcripts (all categories) were mapped to genome scaffolds. The proportion of hits from 84,471 in each category is shown in brackets.

Thirdly, the 87,612 bark transcripts were validated by using the Exonerate software to map them to rubber genome scaffolds (BioProject ID: PRJNA80191). Figure 2 shows the number of bark transcripts which were mapped to categories of transcript-to-scaffold coverage. The higher the percentage of transcript-to-scaffold coverage, the greater the significance of match alignment. A large proportion of bark transcripts (84,471 or 96.41% of total) could be mapped to the scaffolds and of these, almost 70% showed transcript-to-scaffold coverage of 90-100% (Figure 2). Therefore, this indicated the presence of a sizeable proportion of high quality bark transcripts.

As a whole, results of the three mapping analyses carried out provided sufficient validation for the quality of 87,612 bark transcripts assembled from the 16 Gb read set. Fragmented or erroneous transcripts could still be present to some extent in any assembly but we think that the proportion of bark transcripts which did not show meaningful mapping or alignment with *Hevea* transcripts or genome scaffolds could also be explained by reasons such as inherent variations between sequences derived from different tree clonal varieties.

### Mapping saturation test for bark transcript accumulation

**Figure 3:**
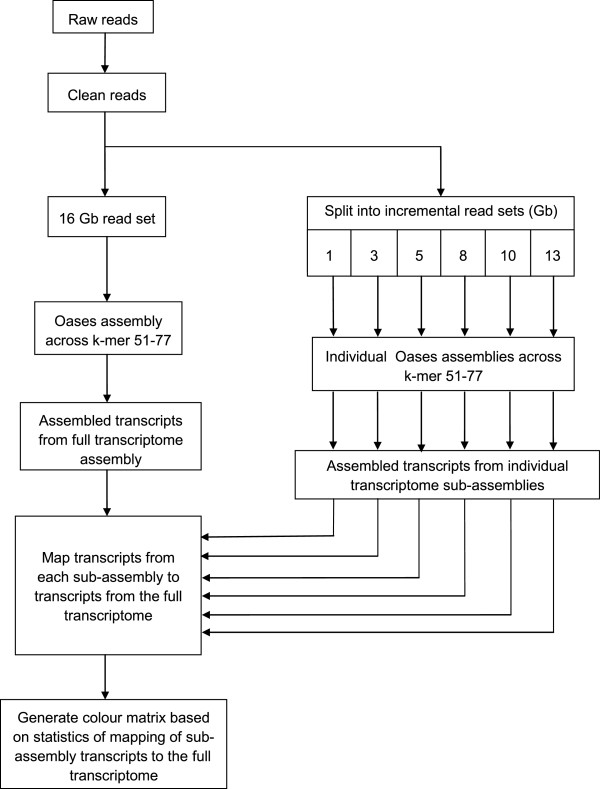
Methodology of the transcript mapping saturation test.

Next, we addressed the question of read depth requirement by mapping the series of incremental bark transcripts to total transcripts from the optimized 16 Gb bark assembly. The principle behind mapping subsets of bark transcripts to total transcripts from the full 16 Gb bark transcriptome is that the number of the former aligning to the latter should follow a saturation pattern. As outlined in Figure 3, transcript sets from 1, 3, 5, 8, 10 and 13 Gb bark reads that had been assembled independently across k-mers 51–77 were mapped to 87,612 bark transcripts. The extent of transcript mapping, expressed as a percentage of total bark transcripts (or the full transcriptome) was taken as a measure of transcript representation by each sub-assembly.

**Figure 4:**
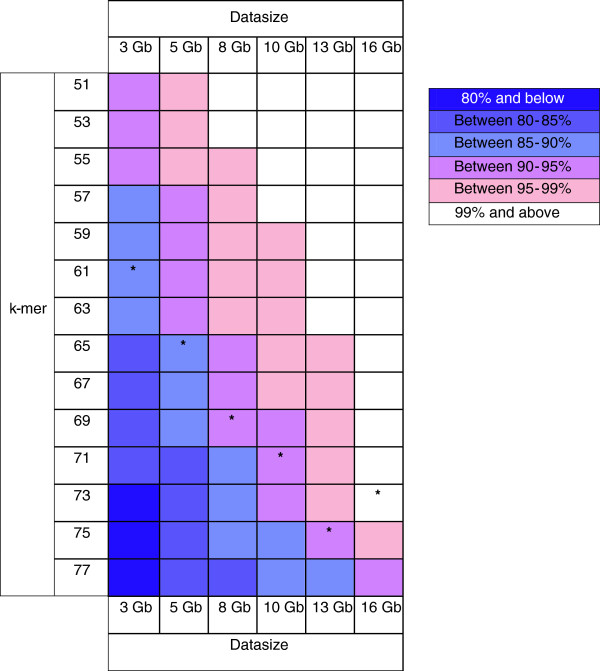
**Colour matrix representing the transcript mapping saturation test results.** An asterisk corresponds to the assembly with the optimized k-mer and transcript N50 length for a particular datasize (1 Gb assembly transcripts were not included since the transcript N50 length was not optimized by any k-mer for this datasize). See Additional file 2: Table S2 for full details of BlastN matches by subsets of bark transcripts to the optimized 16 Gb bark transcriptome.

Results of the number of mappings to 87,612 bark transcripts were displayed as a colour matrix as shown in Figure 4 and Additional file 2: Table S2. Overall, transcript mapping by incremental subsets of bark transcripts approached full saturation (i.e. 100%) as datasize increased (for any k-mer) or as k-mer decreased (for any datasize). Transcript representation was high in all cases, being more than 80% transcript mapping level with the exception of three higher k-mer assemblies (73–77) of 3 Gb reads (Figure 4 and Additional file 2: Table S2). At the lower end of the saturation spectrum, 3–8 Gb reads would already be sufficient to obtain even 80-85% mapping level or transcript coverage. This datasize range could also readily generate transcript coverage exceeding 90% if the k-mer range for assembly was decreased. At the upper end of the saturation spectrum, transcripts from nearly all of the 10–13 Gb assemblies across all k-mers showed mapping levels greater than 90%.

However, although the colour matrix indicated generally high transcript coverage by assemblies of 3–13 Gb reads, it is important to select a datasize that would also produce the optimal transcript N50 length at the desired transcript coverage level. As determined previously, the optimized N50 increased with datasize (Table 3). In the optimized 3, 5, 8, 10 and 13 Gb assemblies, this corresponded with 87.21, 89.48, 91.46, 92.60 and 92.12% representation of the 16 Gb bark transcripts respectively (Additional file 2: Table S2). Therefore, based on the colour matrix, the optimized 3–5 Gb assemblies would fall within the 85-90% transcript coverage bracket and the optimized 8–13 Gb assemblies within the 90-95% coverage bracket (Figure 4 and Additional file 2: Table S2). This also indicated that based on the mapping saturation test in this study, a shift in bark transcript coverage bracket by the incremental assemblies occurred between 5–8 Gb.

In essence, the amount of reads for optimal coverage of a tissue transcriptome should take into consideration the requirements for transcript coverage level (reflected by percentage of mapping saturation) and for N50 length. In order to attain both high transcript representation and best N50 length, our general recommendation for *Hevea* is to generate between 5–8 Gb reads for *de novo* assembly. Firstly, as observed in the shift in transcript coverage bracket, a minimum of nearly 90% (i.e. 89.48%) transcript coverage could already be achieved by an optimized 5 Gb assembly (Figure 4 and Additional file 2: Table S2), a level that is within range of the following coverage bracket (90-95%). Secondly, it is noted from Table 3 that the improvement in optimized N50 length was more rapid from 3–8 Gb than from 8–16 Gb assemblies. Therefore, generating less than 5 Gb reads may lead to reduction in complete transcripts, and sequencing beyond 8 Gb reads may not yield significantly more new or complete transcripts other than the rarely expressed ones.

### Analysis of latex and leaf transcriptome assembly

RNAs from different types of tissues and growth conditions are of interest in *Hevea* transcriptome profiling studies. Application of the recommended 5–8 Gb sequencing depth for *Hevea* would assume the assembly trends to be the same between reads generated from bark and from other tissues. To validate this, we performed Oases assembly of 5 Gb latex and 5 Gb leaf read sets using the same parameters as those for bark assembly (see Materials and methods). Assemblies were performed in incremental read amounts of 1, 3 and 5 Gb across a k-mer range of 51–77. Table 5 shows the statistics of latex and leaf assemblies which correspond to the same quantums of read assemblies in Table 3. As with bark assembly, the transcript N50 length showed a peaking trend only in the 3 and 5 Gb leaf read assemblies across the k-mer range but not in the 1 Gb assembly. On the other hand, the transcript N50 length showed a peak for all three latex assemblies across the k-mers. For the 5 Gb datasize assemblies, the optimized N50 was highest for bark (1,900 bp) followed by latex (1,281 bp) and leaf (1,086 bp) (Tables 3 and 5). Differences in statistics such as total assembled transcripts and optimized N50 values could be due to the presence of tissue-specific genes and variation in dynamic range of transcript levels in tissues, all of which have effect on the outcome of assembly. However, similar to that for bark, the optimized N50 of latex and leaf assemblies increased with read amounts. In conclusion, assembly of reads generated from different tissues did not display major or unexplainable differences between one another, and therefore, the mapping saturation test should be applicable to other *Hevea* tissues.

**Table 5:** **Statistics of 1**, **3 and 5 Gb assemblies of latex and leaf reads**

**k-mer size**	**Latex 1 Gb**		**Latex 3 Gb**		**Latex 5 Gb**		**Leaf 1 Gb**		**Leaf 3 Gb**		**Leaf 5 Gb**	
	**N50 (bp)**	**Total transcripts**	**N50 (bp)**	**Total transcripts**	**N50 (bp)**	**Total transcripts**	**N50 (bp)**	**Total transcripts**	**N50 (bp)**	**Total transcripts**	**N50 (bp)**	**Total transcripts**
51	706	64,691	1016	89,882	1053	108,246	544	90,842	831	141,047	845	170,274
53	716	61,113	1058	85,854	1135	104,493	535	84,656	888	129,418	920	158,195
55	***724***	56,456	1093	79,303	1187	95,718	523	77,815	915	117,125	999	141,823
57	717	52,339	1102	73,610	1231	88,114	517	70,653	933	105,513	1049	127,329
59	707	48,151	***1108***	67,662	1266	80,620	509	64,286	***937***	95,662	1079	113,686
61	683	43,857	1100	62,627	***1281***	73,298	500	58,367	933	86,244	***1086***	100,912
63	668	40,003	1090	57,318	1270	67,227	490	52,086	912	78,666	1084	90,894
65	657	36,366	1067	53,173	1266	61,105	478	46,805	885	72,173	1059	82,529
67	642	32,702	1041	49,168	1241	55,834	466	41,495	855	66,289	1026	75,410
69	629	29,326	1001	46,360	1201	52,592	451	36,757	810	61,116	988	68,544
71	610	26,079	956	43,095	1151	49,302	439	32,167	761	56,152	944	63,072
73	591	23,055	907	40,051	1103	45,844	432	27,547	713	51,241	879	58,710
75	578	20,055	850	36,849	1052	42,945	427	23,246	669	46,727	823	53,571
77	567	17,326	815	33,398	990	39,996	427	19,079	631	41,838	763	49,712

The optimized N50 length for each tissue assembly is highlighted in bold italics.

## Discussion and conclusions

Knowing whether transcriptome sequencing and assembly have substantially captured all the genes expressed in a sample is an important consideration for plants having limited genomic resources as reference. Generally, the amount of reads for comprehensive coverage of a *de novo* transcriptome is often determined by a balance of budget, capacity of sequencing platform and guesstimates or “best practices” based on other species. In this work, we report a systematic approach which we name the “transcript mapping saturation test” to assess the amount of reads required for optimal transcriptome coverage in the *Hevea* rubber tree. This was made possible by the availability of 16 Gb Illumina PE RNA-Seq reads from *Hevea* bark which enabled us to map transcripts from incremental sub-assemblies to transcripts from the entire assembly (or the full transcriptome) in order to detect the mapping saturation point. The workflow of this methodology is outlined in Figure 3, beginning with assembly optimization and validation of the full transcriptome, followed by the mapping saturation test.

Because sequencing has become increasingly affordable, obtaining as much as 16 Gb reads per sample as a starting point is not insurmountable. Using our approach, sequencing to this extent has to be done only once in the beginning, after which the user is equipped with a guide (the colour matrix) to estimate optimal coverage of expressed genes in the plant species of interest. In developing this approach, we used the Oases assembler because Velvet, for which Oases is an extension, had previously been found to be suitable for producing quality transcripts from *Hevea* short reads [29]. Thus, we progressed to Oases, which additionally has the ability to resolve alternatively spliced transcripts [5]. We would suggest that a *de novo* project intending to adopt our approach should first test if the assembler of choice is suited to their transcriptome. Even though our method development is based on Illumina PE RNA-Seq reads, the principle behind this approach should also be applicable to other plant species and to reads from other sequencing platforms.

Although the transcript mapping approach is based on mapping saturation, this does not reduce the need to validate the assembly quality of the full transcriptome. In this work, the N50 trend was used in the initial selection of best k-mer for assembly. Subsequently, the completeness and correctness of assembled transcripts were supported by results of mapping to rubber genome scaffolds [60] whereby a significant proportion showed transcript-to-scaffold coverage of 90% and above. This was also supported by detection of all but one of 248 core genes expressed in eukaryotes [66] and significant alignments with known *Hevea* protein coding frames. However, we should point out that what this paper proposes is essentially a methodology; we do not specifically assert that a 5–8 Gb read depth would be sufficient for optimal transcriptome coverage universally. The optimal read depth may differ in other species depending on factors such as genome size and transcript complexity.

## Materials and methods

### Plant material and RNA isolation

Latex and bark shavings were obtained from 15-year old RRIM 928 *Hevea brasiliensis* trees growing in the Rubber Research Institute of Malaysia Research Station, Sungai Buloh. Equal volumes of latex were tapped from three trees and collected directly into 2× RNA extraction buffer [67]. The bark just below the tapping cut of the trees was scraped to remove surface matter before bark shavings (approximately 1 cm depth) were excised with a tapping knife. Young leaves of RRIM 928 trees were collected from the source bush nursery in the Rubber Research Institute of Malaysia Research Station, Sungai Buloh.

Total RNA was isolated from latex and leaf tissues using the phenol-chloroform method [67]. Bark total RNA was isolated using a modified procedure of the Qiagen RNeasy Plant Mini Kit [68]. RNA samples were assessed for quality and quantity using the Nanodrop spectrophotometer (Thermo Scientific).

### Sequence generation and quality assessment

Bark, latex and leaf total RNAs (20 μg each) were sent to the Illumina Fast Track sequencing service in San Diego, USA where 200 bp fragment size libraries were produced for paired-end RNA sequencing (PE RNA-Seq). Each RNA-Seq sample was sequenced 100 nucleotides at each end (2 × 100 nt), resulting in about 50 million raw reads each from latex and leaf and nearly 170 million raw reads from the bark (Table 2). Raw reads from bark, latex and leaf are available from the NCBI Sequence Read Archive (accession nos. SRX278513-5).

Clean reads were obtained by trimming raw reads at a minimum phred score of Q = 20, followed by removal of reads below 30 bp and subsequently reads which contained ‘N’ nucleotides. Clean paired reads from the bark (163,316,702 reads; see Table 2) were referred to as the 16 Gb read set and clean paired read sets from the latex and leaves (48,650,932 and 46,062,766 reads respectively; see Table 2) as the 5 Gb read sets. These read sets were used for subsequent transcriptome assembly. Clean paired reads were classified into arbitrary nucleotide size categories to confirm good PE RNA-Seq data quality (Figure 1).

### Transcriptome assembly and transcript mapping

Clean paired reads from the bark, latex and leaf (Table 2) were assembled with the Velvet (Version 1.1.05) [4] and Oases assembler (Version 0.1.22) [5] using default parameters and selection of a minimum transcript length of 100 bp. A range of hash lengths (k-mers 51–77) was used for assembly of a read set to determine the k-mer which produced the highest transcript N50 length. This best N50 value was termed as the “optimized N50” while the hash length which produced it was the “optimized k-mer”. Note: N50 length is the length of the shortest transcript whereby the sum of transcripts of equal length or longer is at least 50% of the total length of all transcripts.

Incremental quantums of bark reads (1, 3, 5, 8, 10, 13 and 16 Gb) were obtained by partitioning the subsets from the 16 Gb read set. Each subset was random as the 16 Gb read set was already fully randomized. (Similarly, 5 Gb read sets from latex and leaf were also fully randomized). Serial mapping of bark transcripts was performed using BlastN [69] and the top hit by any query transcript with e-value ≤ 1.0e^-5^ was counted as a match. The complete methodology for the transcript mapping saturation test is shown in Figure 3.

### Bark transcript validation

For evaluation of rubber-specific ORF quality of 87,612 bark transcripts from the 16 Gb assembly (k-mer 73), 255 *Hevea* sequences which were confirmed to encode complete ORFs were selected from the NCBI GenBank non-redundant database (http://www.ncbi.nlm.nih.gov). These *Hevea* cDNAs, which were isolated by traditional gene cloning approaches such as cDNA library hybridization and PCR, were generally of high quality as they had mainly been obtained by Sanger sequencing (see Additional file 1: Table S1). Megablast [69] was used to map the 255 *Hevea* ORFs to 87,612 transcripts from the 16 Gb bark assembly. Top hits from this analysis (with 86-100% sequence identity match) were screened for high quality matches based on a minimum of 70% coverage of *Hevea* ORFs (or query coverage) in their alignments to bark transcripts (the subject) (see Table 4 and Additional file 1: Table S1).

For evaluation of completeness of assembled bark transcripts, 248 core eukaryotic genes (CEGs) of *Arabidopsis thaliana* were downloaded from the CEGMA resource at http://korflab.ucdavis.edu/Datasets/genome_completeness/. This approach was based on a list of 248 highly conserved but low copy number genes that had been shown to be a reliable indicator of completeness of gene space in eukaryotic species [66]. Using BlastX [69], 87,612 bark transcripts were mapped to the CEGs with any hit of e-value ≤ 1.0e^-10^ counted as a match.

Rubber genome scaffolds from the BioProject ID: PRJNA80191 (http://www.ncbi.nlm.nih.gov/nuccore/448814761) were used for validating bark transcripts. Bark transcripts were mapped to genome scaffolds by Exonerate (Version 2.2.0) [70] using default settings with the exception of the following parameters: heuristic mode, est2genome model and alignment score of at least 10 percent of the maximal score for each query. The significance of mapped transcripts was evaluated by calculating query coverage which is expressed as percentage of the transcript sequence (query) that overlaps with the scaffold sequence (subject). This percentage reflects the extent of bark transcript coverage in alignments to genome scaffolds. Scaffold hits were classified according to transcript coverage whereby the higher the percentage, the greater the significance of transcript-to-scaffold alignment (see Figure 2).

## Availability and requirements

The datasets, SRX278513-5, supporting the results of this article are available in the NCBI Sequence Read Archive:

http://www.ncbi.nlm.nih.gov/sra/?term=SRX278513

http://www.ncbi.nlm.nih.gov/sra/?term=SRX278514

http://www.ncbi.nlm.nih.gov/sra/?term=SRX278515

## Competing interests

The authors declare that they have no competing interests.

## Authors’ contributions

A-KG, C-CH and K-SC conceptualized the transcript saturation mapping methodology. A-KG and C-CH performed the transcriptome assembly, transcript mapping and other bioinformatics analysis. K-SC wrote the manuscript with contributions from A-KG and C-CH during final editing. K-SC and ZMZ initiated the tissue transcriptome sequencing project including the selection of tree material, RNA preparations and other supporting analyses. All authors approved the final manuscript.

## Supplementary Material

Additional file 1: Table S1 Megablast analysis of 255 *Hevea* complete ORFs against 87,612 bark transcripts from the optimized 16 Gb bark assembly. The last 5 rows show *Hevea* ORFs which did not have any Megablast hit. Percentage identity of alignments in hits by 250 *Hevea* ORFs ranged from 86-100% (see column J). Highlighted rows (total of 50) indicate hits which showed less than 70% utilization of ORF sequence length in their alignments (see column K). Click here for file

Additional file 2: Table S2 Colour matrix (full version) representing the transcript mapping saturation test results. BlastN matches (e-value ≤ 1.0e^-5^) by subsets of bark transcripts to 87,612 transcripts from the optimized 16 Gb bark assembly are shown as numbers and percentages (in brackets) of the total. Numbers in bold correspond to the assembly with the optimized k-mer and transcript N50 length for a particular datasize. The 1 Gb assembly transcripts are not included since transcript N50 length was not optimized by any k-mer for this datasize. Click here for file
