# Supplementary material for: RNA sequencing read depth requirement for optimal transcriptome coverage in Hevea brasiliensis
Source: BMC Res Notes. 2014 Feb 1;7:69. doi: 10.1186/1756-0500-7-69 (PMC3926681; doi:10.1186/1756-0500-7-69)
Supplement: Additional file 2: Table S2 — Colour matrix (full version) representing the transcript mapping saturation test results. BlastN matches (e-value ≤ 1.0e-5) by subsets of bark transcripts to 87,612 transcripts from the optimized 16 Gb bark assembly are shown as numbers and percentages (in brackets) of the total. Numbers in bold correspond to the assembly with the optimized k-mer and transcript N50 length for a particular datasize. The 1 Gb assembly transcripts are not included since transcript N50 length was not optimized by any k-mer for this datasize. [file 1756-0500-7-69-S2.docx]

|  | Datasize | | | | | |
| --- | --- | --- | --- | --- | --- | --- |
| k-mer | 3 Gb | 5 Gb | 8 Gb | 10 Gb | 13 Gb | 16 Gb |
| 51 | 81,906  (93.49%) | 85,270  (97.33%) | 87,049  (99.36%) | 87,399  (99.76%) | 87,515  (99.89%) | 87,558  (99.94%) |
| 53 | 80,940  (92.38%) | 84,665  (96.64%) | 86,846  (99.13%) | 87,327  (99.67%) | 87,501  (99.87%) | 87,555  (99.93%) |
| 55 | 79,936  (91.24%) | 83,963  (95.84%) | 86,523  (98.76%) | 87,161  (99.49%) | 87,484  (99.85%) | 87,558  (99.94%) |
| 57 | 78,757  (89.89%) | 83,070  (94.82%) | 86,092  (98.27%) | 86,967  (99.26%) | 87,427  (99.79%) | 87,564  (99.95%) |
| 59 | 77,621  (88.60%) | 82,037  (93.64%) | 85,527  (97.62%) | 86,671  (98.93%) | 87,348  (99.70%) | 87,548  (99.93%) |
| 61 | **76,410**  **(87.21%)** | 80,901  (92.34%) | 84,785  (96.77%) | 86,226  (98.42%) | 87,199  (99.53%) | 87,552  (99.93%) |
| 63 | 75,233  (85.87%) | 79,727  (91.00%) | 83,987  (95.86%) | 85,677  (97.79%) | 87,007  (99.31%) | 87,539  (99.92%) |
| 65 | 73,969  (84.43%) | **78,396**  **(89.48%)** | 82,904  (94.63%) | 84,871  (96.87%) | 86,710  (98.97%) | 87,512  (99.89%) |
| 67 | 72,740  (83.03%) | 77,017  (87.91%) | 81,592  (93.13%) | 83,884  (95.74%) | 86,206  (98.40%) | 87,500  (99.87%) |
| 69 | 71,573  (81.69%) | 75,562  (86.25%) | **80,133**  **(91.46%)** | 82,620  (94.30%) | 85,514  (97.61%) | 87,493  (99.86%) |
| 71 | 70,541  (80.52%) | 74,222  (84.72%) | 78,550  (89.66%) | **81,125**  **(92.60%)** | 84,583  (96.54%) | 87,498  (99.87%) |
| 73 | 69,397  (79.21%) | 72,926  (83.24%) | 76,855  (87.72%) | 79,466  (90.70%) | 83,357  (95.14%) | **87,604**  **(99.99%)** |
| 75 | 68,163  (77.80%) | 71,670  (81.80%) | 75,193  (85.83%) | 77,413  (88.36%) | **80,709**  **(92.12%)** | 84,153  (96.05%) |
| 77 | 66,591  (76.01%) | 70,432  (80.39%) | 73,745  (84.17%) | 75,590  (86.28%) | 78,286  (89.36%) | 81,178  (92.66%) |
| k-mer | 3 Gb | 5 Gb | 8 Gb | 10 Gb | 13 Gb | 16 Gb |
|  | Datasize | | | | | |

| 80% and below |
| --- |
| Between 80-85% |
| Between 85-90% |
| Between 90-95% |
| Between 95-99% |
| 99% and above |
|  |
